# Degradation of limonene and *trans*-cinnamaldehyde in soil, and detection of their metabolites by UHPLC and GC-HRMS

**DOI:** 10.1007/s11356-024-33334-6

**Published:** 2024-04-26

**Authors:** Alba Reyes-Ávila, Antonia Garrido Frenich, Roberto Romero-González

**Affiliations:** Research Group “Analytical Chemistry of Contaminants”, Department of Chemistry and Physics, Research Centre for Mediterranean Intensive Agrosystems and Agrifood Biotechnology (CIAMBITAL), Agrifood Campus of International Excellence (ceiA3), University of Almeria, 04120 Almeria, Spain

**Keywords:** Commercial biopesticides, Limonene, *trans*-Cinnamaldehyde, Soil, Metabolites, HRMS

## Abstract

Two commercial biopesticides were studied to determine their persistence in two soil types, such as sandy clay loam and clay loam soils. For this purpose, an orange oil–based biopesticide was used, being limonene its main ingredient. The other biopesticide was based on cinnamon extract and *trans*-cinnamaldehyde as its main component. Degradation of these compounds was monitored, and transformation products or metabolites were detected. Limonene and its metabolites were analyzed by gas chromatography (GC) and *trans*-cinnamaldehyde by ultra-high-performance liquid chromatography (UHPLC). Both techniques were coupled to a high-resolution mass (HRMS) analyzer, such as quadrupole (Q)-Orbitrap. Limonene and *trans*-cinnamaldehyde were rapidly degraded as result of first-order kinetics. Possible metabolites such as thymol, cymene, isoterpinolene and cymenene for limonene, and hydroxycinnamic acid for *trans*-cinnamaldehyde were tentatively identified. Moreover, four other metabolites of *trans*-cinnamaldehyde, some of them not previously described, were also detected.

**Supplementary Information:**

The online version contains supplementary material available at 10.1007/s11356-024-33334-6.

## Introduction

In recent years, less toxic pesticides have been used to reduce the potential risk for environmental contamination, such as soil and water. It also minimizes the risk to human health and does not alter the soil microbiome, which is critical to the proper functioning of the environment (Rajmohan et al. 2020). For this purpose, natural pesticides based on minerals, plants, or microorganisms, known as biopesticides, have been developed (US EPA 2022). The use of plant extracts or essential oils against various pests has been carried out since ancient times (Haritha et al. 2021), proving its effectiveness against different types of insects (Cárdenas-Ortega et al. 2015; da Silva et al. 2023). These extracts usually contain a high level of volatile compounds, such as monoterpenes and other volatile analytes. Among plant-based biopesticides, those derived from essential oils such as pyrethrins or azadirachtin stand out (Fenibo et al. 2022). Additionally, limonene and *trans*-cinnamaldehyde are monoterpenes whose properties as insecticides have been studied (Denkova-Kostova et al. 2021; de Andrade Rodrigues et al. 2022). Therefore, several commercial biopesticides based on extract plants have been manufactured, where these two compounds are present at high concentration.

Despite their growing use, biopesticides make up only 5% of the global pesticide market (Kumar et al. 2021; Fenibo et al. 2022), but it is expected that annual growth will reach 8% by 2023 (Yadav et al. 2022). One of the circumstances that prevents the expansion of the use of biopesticides is the strict restrictions that are applied before they are marketed. This prevents the development of new biopesticides that may be commercialized. In the United States of America (USA) or China, restrictions are less strict than in the European Union (EU) (Kumar et al. 2021). As a result, there are only 60 to 80 biopesticides registered in the EU, compared with 200 to 400 in the USA (Kumar et al. 2021; Fenibo et al. 2022), and in the global market, the 63% of commercially available biopesticides are microbial biopesticides.

Synthetic pesticides have been investigated for their persistence in the environment (Zhou et al. 2022; Merlo-Reyes et al. 2024), as well as the metabolites or transformation products of their active principles during the degradation process (Vargas-Pérez et al. 2020; López-Ruiz et al. 2020). Despite the growing expansion of biopesticides, studies of their degradation in the environment are limited (López-Serna et al. 2016; Huang et al. 2022). Most studies on biopesticides in soil and/or in water focus on azadirachtins (Prestes et al. 2012; Suciu et al. 2019) and pyrethrins (Prestes et al. 2012; Feng et al. 2018). In these studies, the extraction methods commonly used to extract them are QuEChERS (acronym of Quick, Easy, Cheap, Effective, Rugged, and Safe) (Prestes et al. 2012; Feng et al. 2018; Suciu et al. 2019). In addition, they use gas chromatography (GC) (Feng et al. 2018), although high-performance liquid chromatography (HPLC) (Prestes et al. 2012; Suciu et al. 2019) can also be utilized. As detectors, quadrupole (Q) (Feng et al. 2018) for GC and triple quadrupole (QqQ) (Prestes et al. 2012) or diode-array detector (DAD) (Suciu et al. 2019) for UHPLC are commonly employed.

However, there are few studies on the extraction of limonene and *trans*-cinnamaldehyde in soil (López-Serna et al. 2016; Huang et al. 2022). For *trans*-cinnamaldehyde, a previous study only examined the mobility of the compound in soil (López-Serna et al. 2016) and did not evaluate its degradation and metabolites. On the other hand, only one study monitored the degradation of limonene and its metabolites in the soil but low-resolution mass spectrometry was utilized (Huang et al. 2022). Therefore, a study was carried out to monitor the degradation of limonene and *trans*-cinnamaldehyde in several soil types. UHPLC has been used to monitor *trans*-cinnamaldehyde, and GC for limonene, and most of these previous studies have employed low-resolution mass analyzers such as Q (López-Serna et al. 2016; Huang et al. 2022). Bearing in mind these previous studies, an innovation in this study is the use of high-resolution mass spectrometry (HRMS) using a Q-Orbitrap analyzer to monitor the degradation of both compounds. In addition, possible transformation products or metabolites of these compounds have been analyzed. To do this, an untargeted analysis has been carried out using suspect and unknown modes. Thus, understanding the fate of these metabolites provides a more comprehensive insight into the true impact of these biopesticides on the soil, enabling the collection of data regarding their potential toxicity and permanence in the soil.

## Materials and methods

### Materials

Two commercial biopesticides, Cinna (Hortalan; El Ejido, Spain) and Prevam® (ORO AGRI; Palmela, Portugal), based on cinnamon extracts and orange essential oil respectively, were obtained.

Ethyl acetate (EtOAc, ≥99.7%) and methanol (MeOH, ≥99.9%) were provided from Honeywell (Charlotte, NC, USA), whereas formic acid (99.0%) and water (H_2_O, LiChrosolv®) were from Merck (Darmstadt, Germany). All solvents were HPLC grade.

Analytical standards used were thymol provided by Tokyo Chemical Industry (Tokyo, Japan), (R)-(+)-limonene and m-cymene by Sigma Aldrich (Saint Louis, MO, USA), and *trans*-cinnamaldehyde by Dr. Ehrenstorfer (Augsburg, Germany). Internal standards (IS) were triphenyl phosphate provided by Supelco (Darmstadt, Germany) for UHPLC and biphenyl (Dr. Ehrenstorfer) for GC.

For each compound, individual stock solutions were prepared at 1000 mg/L in EtOAc. From the stock solutions, individual intermediate solutions at 10 and 1 mg/L in EtOAc were made. These solutions were kept at −18 °C.

Extracts were filtered with an Econofltr nylon filter 0.2 µm, 13 mm (Agilent Technologies; Santa Clara, CA, USA).

### Equipment

UHPLC and GC methods used were optimized in a previous study (Reyes-Ávila et al. 2023).

#### UHPLC method

A Vanquish™ Flex Quaternary LC (Thermo Fisher Scientific; Waltham, MA, USA) was the chromatographic equipment with a C18 Hypersil GOLD™ aQ column (2.1 x 100 mm, 1.9 µm) purchased by Agilent. Mass spectrometer was a Q-Exactive Orbitrap, provided by Thermo Fisher.

Electrospray interface (ESI) has been used with a collision energy of 30 eV (higher-energy collisional dissociation, HCD). The acquisition mode used was full scan (74–1100 *m*/*z* range) with a resolution of 70,000 full width at half maximum (FWHM). The automatic gain control (AGC) value was equal to 10^6^. Data-dependent acquisition (DDA), in negative and positive ionization modes, was used. DDA resolution was 35,000 FWHM, and AGC value was set at 10^5^. Minimum AGC target value was 8·10^3^. The flow rate was 0.2 mL/min, the injection volume was set at 10 μL, and the column temperature was 30 °C. The mobile phase consisted of MeOH as organic phase and an aqueous solution of formic acid (0.1%) as aqueous phase. The gradient mode started with a constant composition of 5 % MeOH during 2 min. Then, it was increased up to 100% MeOH during 14 min, and this composition was kept constant from 16 to 26 min. Finally, the composition decreased to 5% MeOH in 1 min, and it was kept constant for 3 min to equilibrate the column. Total running time was 30 min. The ESI conditions were as follows: auxiliary and sheath gas used, N_2_ (95%); heater temperature, 305 °C; capillary temperature, 300 °C; spray voltage, 4 kV; and the S-lens radiofrequency level was 50 (arbitrary units).

#### GC method

A TRACE™ 1310 GC system was the chromatographic equipment with a TriPlus™ RSH autosampler (Thermo Scientific) and a J&W DB-5ms non-polar column (30 m × 0.25 mm × 0.25 μm) from Agilent Technologies, coupled to a Q-Exactive Orbitrap (Thermo Fisher Scientific) mass spectrometer. The injection volume was 1 µL. For chromatographic conditions, initial oven temperature was 60 °C (hold 2 min) and it was increased at 6 °C/min rate to 220°C (hold 2 min). Finally, it was raised to 280 °C with a 20 °C/min rate (hold 4 min). The total running time was 37 min. For MS conditions, full scan in positive mode was used (30–450 *m*/*z* range) with a 70-eV positive electron ionization (EI). The resolution was 70,000 FWHM, and an AGC value was set 10^6^. Helium was used as carrier gas with a constant flow rate of 1 mL/min.

### Soil samples

Four different soils have been used: two sandy clay loam soils (SCL1 and SCL2) and two clay loam soils (CL1 and CL2). The soils were collected in several greenhouses located in Roquetas de Mar, El Ejido and Vícar, which are placed in the southeast of Spain (Almeria). Before analysis, the soil was dried at ambient temperature for three days and sifted to a particle size < 2 mm. Their physicochemical information is collected in Table S1.

### Laboratory studies

Degradation studies were performed in the research group’s laboratory. The experiments were carried out at room temperature (20 ºC) and with natural sunlight (8 h of light).

First, aliquots (20 g) of each soil (SCL1, SCL2, CL1, and CL2) were weighed in Erlenmeyer flasks. To mimic soil humidity conditions, water (6 and 3 mL) was added to clay loam soils (30% humidity) and to sandy clay loam soils (15% humidity), respectively. Different sampling times were selected: 0 h, 4 h, 1, 1.5, 2, 3, 4, 7, and 9 days. In both SCL1 and CL1 soils, an application rate according to the manufacturer’s recommendations (8 L/ha, normal dose rate) and twice the recommended dose (16 L/ha, double dose rate) of the commercial biopesticide Prevam® were applied. On the other hand, in SCL1, SCL2, and CL2 soils, a normal dose rate (300 mL/hL) and a double dose rate (600 mL/hL) of the commercial biopesticide Cinna were applied. The highest application rate was used to improve the detection of possible metabolites. To prepare the dose rates, the commercial biopesticides were diluted in water until reaching the desired dose. The theoretical normal dose rate of limonene and *trans*-cinnamaldehyde, which were previously characterized (Reyes-Ávila et al. 2023), was 2377 µg/kg and 8477 µg/kg, respectively. Every 2 days, water was added to restore its loss in each Erlenmeyer. Three replicates were made for each type of soil and time.

### Extraction method

The extraction of biopesticides from soil was carried out using a solid-liquid extraction. For this, soil samples (5 g) was weighed in 50 mL centrifuge tubes. Then, 100 µg/kg of each IS, biphenyl, and triphenyl phosphate for GC and UHPLC, respectively, was added. After that, 10 mL EtOAc was added. The sample was put on a rotary shaker for 1 h. Afterwards, the mixture was centrifuged at 5000 rpm for 5 min. Finally, they were filtered. Three replicates of each sample were made. Limonene was analyzed by GC-Q-Orbitrap and *trans*-cinnamaldehyde by UHPLC-Q-Orbitrap.

### Method validation

For the validation of the extraction method using UHPLC-Q-Orbitrap and GC-Q-Orbitrap, limits of detection (LOD) and quantification (LOQ), linearity, and matrix effect were calculated. Moreover, intra-day precision (repeatability) and trueness (recovery, %) were evaluated.

LODs and LOQs were calculated by injecting enriched blank samples at low concentrations between 1 and 50 µg/kg. The coefficients of determination (*R*^2^) from the calibration curves (1–250 μg/L) were used to calculate the linearity. The matrix effect was measured by studying standards prepared in an extracted blank soil matrix and standards in EtOAc, which ranged from 1 to 250 μg/L. Precision was determined by carrying out a repeatability study. The relative standard deviation (% RSD) for each analyte was expressed with five replicates at each concentration level (10 and 100 µg/kg). Trueness was studied by analyzing samples spiked at 10 and 100 µg/kg with five replicates for each concentration.

### Data analysis

Data were processed using Xcalibur 3.0, with QualBrower and QuanBrowser. For the analysis of metabolites, Compound Discoverer™ 3.3 program (Thermo Fisher Scientific) and MassChemSite 3.1 (Mass Analytica, Sant Cugat del Vallés, Spain) were employed. Moreover, National Institute of Standards and Technology (NIST) MS Search 2.2 library has been utilized.

For metabolite untargeted analysis, the parameters chosen for Compound Discoverer were 0.1 min (retention time tolerance), 0.1% (intensity threshold), 3 (S/N threshold), 30% (intensity tolerance), 50,000 (min peak intensity), and 5 ppm (mass tolerance). GC-Orbitrap libraries, such as Contaminants Library, Other Environments, PCBs, and Pesticides, and NIST library, such as replib, NISTDEMO, and mainlib, were selected in GC workflow. For UHPLC workflow, the libraries selected were mzVault, mzCloud, and Mass Lists such as the EFS HRAM Compound Database, Lipid Maps Structure Database, Natural Products Atlas 2020_06 or LCMS Co-formulant PPP, and ChemSpider. The selected adducts were [M-H]^−^, [M+H]^+^, [M-H+FA]^−^, [M+Na]^+^, and [M+H-H_2_O]^+^.

The degradation kinetics of limonene and *trans*-cinnamaldehyde in soil was studied using a single first-order rate (SFO) model (Eq.1). To calculate half-life time (DT_50_) and 90% dissipation time (DT_90_), Eqs.2 and 3 was used, respectively,

1 $${C}_{t}={C}_{0}{e}^{-kt}$$

2 $${DT}_{50}=\frac{ln 2}{k}$$

3 $${DT}_{90}=\frac{ln 10}{k}$$

where* C*_0_: concentration at time 0, *C*_*t*_: concentration at a certain time, *t*: time (days); and *k*: rate constant.

## Results and discussion

Limonene and *trans*-cinnamaldehyde have previously been characterized by GC and UHPLC, respectively (Reyes-Ávila et al. 2023). Spectral information for both compounds is shown in Table S2. UHPLC-HRMS was used to monitor the degradation of *trans*-cinnamaldehyde as well as to identify its possible metabolites. Considering limonene was not detected by UHPLC, its degradation was monitored by GC-HRMS.

### Extraction optimization and method validation

The extraction method was optimized by testing several extraction times and procedures with EtOAc as extraction solvent, which was used in previous studies for the extraction of *trans*-cinnamaldehyde (López-Serna et al. 2016). Thus, 5 g of SCL1 was spiked with 50 µg/kg of limonene and *trans*-cinnamaldehyde. Moreover, 50 µg/kg of the corresponding IS was added to each sample. First, the targeted compounds were extracted using as extraction time 30 min and utilizing a rotary agitator. The recoveries obtained for limonene and *trans*-cinnamaldehyde were below the acceptable values (70–120%), being 56.7% and 50.9%, respectively (Table S3). Afterwards, the same procedure was tested, but increasing the extraction time to 1 h. The recoveries obtained were 98.5% (limonene) and 101.4% (*trans*-cinnamaldehyde), and RSD values were 4.7% (limonene) and 1.0% (*trans*-cinnamaldehyde). As the extraction time increased, recovery for both compounds improved within an acceptable range. On the other hand, an attempt was made performing ultrasound-assisted extraction (UAE) for 20 min. The recoveries were 111.6% (limonene) and 111.4% (*trans*-cinnamaldehyde), and RSD values were 4.6% (limonene) and 2.7% (*trans*-cinnamaldehyde). Therefore, it was decided to select the normal extraction for 1 h because it had better recoveries for both compounds and the RSD for *trans*-cinnamaldehyde was lower.

For method validation, the different parameters indicated in the “Method validation” section had been evaluated. The matrix effect was estimated by dividing the slope obtained for limonene and *trans*-cinnamaldehyde, in the solvent by the slope obtained in the matrix for each compound. The matrix effect values were 0.97 for limonene and 0.86 for *trans*-cinnamaldehyde (Table 1). For both compounds, the matrix effect was considered negligible because it was within 0.8 and 1.2. Therefore, the quantification has been carried out with calibration curves prepared in solvent between 20 (limonene)-10 (*trans*-cinnamaldehyde) up to 250 μg/L. Moreover, linearity from the calibration curves was *R*^2^ > 0.991. Recoveries obtained for 10 µg/kg were 83.4% (limonene) and 106.2% (*trans*-cinnamaldehyde), while for 100 μg/kg, they were 100.0% limonene and 93.2% *trans*-cinnamaldehyde. For the repeatability study, RSD ranges from 2.6 to 16.3% for limonene, and 2.8 to 16.4 % for *trans*-cinnamaldehyde were obtained.

**Table 1:** Validation parameters obtained for limonene and *trans*-cinnamaldehyde

Method parameters		Limonene	*trans*-Cinnamaldehyde
Matrix effect^a^		0.97	0.86
*R*^2^		0.999	0.991
LOD (µg/kg)		2	1
LOQ (µg/kg)		10	5
Recovery (%)^b^	10 µg/kg	83.4	106.2
	100 µg/kg	100.0	93.2
Intra-day precision: RSD (%)^b^	10 µg/kg	16.3	16.4
	100 µg/kg	2.6	2.8

Abbreviation: *LOD* limit of detection, *LOQ* limit of quantification, *R*^*2*^ coefficient of determination, *RSD* relative standard deviation

^a^Estimated as the ratio between the slope in matrix and solvent

^b^Number of replicates: 5

### Laboratory studies

**Fig. 1:**
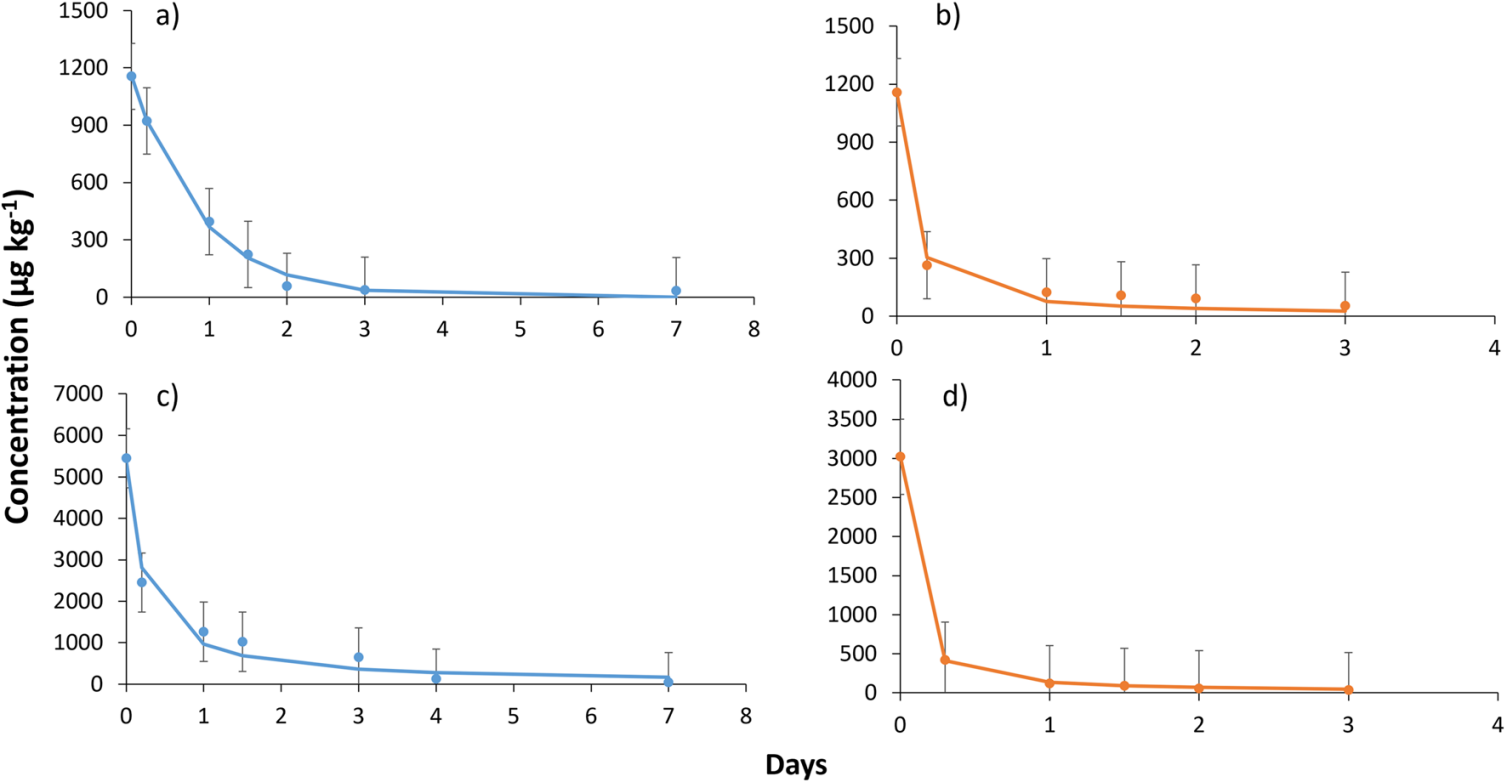
Degradation of limonene at normal dose rate in **a** clay loam soil 1 and **b** sandy clay loam soil 1, and at double dose rate in **c** clay loam soil 1 and **d** sandy clay loam soil 1. Error bars: standard deviation (number replicates = 3)

**Fig. 2:**
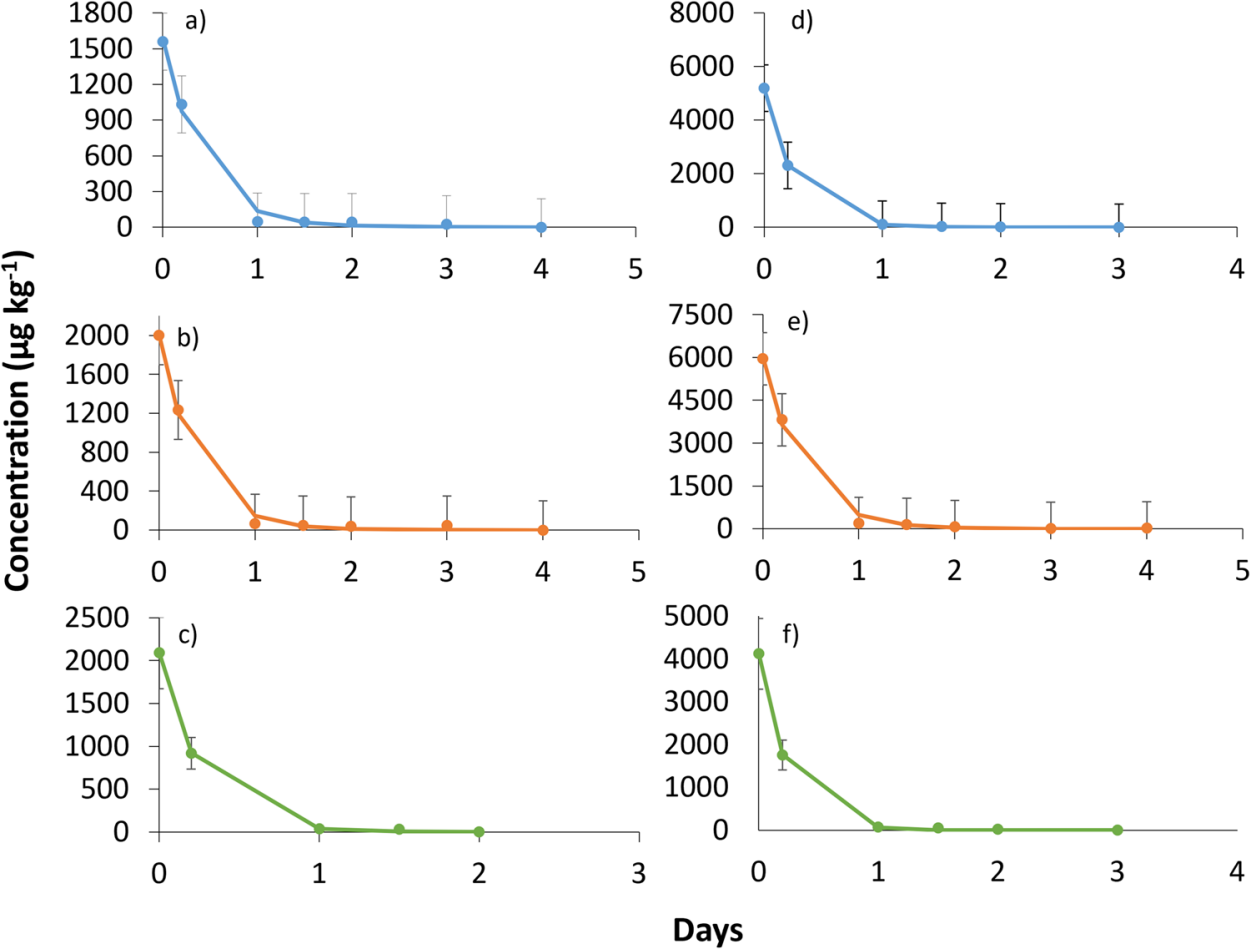
Degradation of *trans*-cinnamaldehyde at normal dose rate in **a** clay loam soil 2, **b** sandy clay loam soil 2, and **c** sandy clay loam soil 1, and at double dose rate in **d** clay loam soil 2, **e** sandy clay loam soil 2, and **f** sandy clay loam soil 1. Error bars: standard deviation (number replicates = 3).

Three replicates of each soil sample spiked with the commercial biopesticide were analyzed at different time intervals, as it was described in the “Laboratory studies” subsection under the “Materials and methods” section. The concentration of limonene and *trans-*cinnamaldehyde varied during the sampling time when using the two dosages (normal and double application rate) for each compound, according to Figs. 1 and 2, respectively.

#### Limonene study

Limonene degradation occurred very quickly in both soil types following a first-order kinetics (Eq**. **1). In CL1 soil, limonene was not detected after 7 days, while in SCL1 soil, it disappeared at 3 days as can be seen in Fig. 1. The DT_50_ values obtained were 0.60 days in CL1 soil and 0.08 days in SCL1 soil at normal dose rate. On the other hand, DT_50_ values at double dose rate have been 0.70 days in CL1 soil and 0.11 days in SCL1 soil as shown in Table 2. In addition, for the CL1 soil, DT_90_ values were 2.00 days (normal dose) and 2.32 days (double dose), while for SCL1 soil, it was 0.28 days (normal dose) and 0.35 days (double dose). These values indicated that limonene was degraded faster in SCL1 soil than in CL1 soil at both doses. In a previous study on limonene in soil, limonene also followed a first-order degradation kinetics, obtaining faster DT_50_ values for the SCL soil type too (Huang et al. 2022). This difference may be attributed to the higher organic matter content in SCL1 soil (4.1%) compared to CL1 soil (1.5%), which serves as sustenance for soil microorganisms (Murphy 2015). Since there was a greater amount of organic matter, it was likely that there is a higher density of microorganisms that degraded limonene faster. In a previous study, the detected oxidation products were also generated by microbial biotransformation (Huang et al. 2022). There are several studies where limonene biotransformation has been investigated by microorganisms and enzymes involved (Tan and Day 1998; van der Werf et al. 1999). Despite the fact there are various microbial biotransformation pathways for limonene, it is also prone to autoxidation due to its relative instability in the presence of oxygen (de Groot 2019).

**Table 2:** Kinetic parameters of limonene and *trans*-cinnamaldehyde degradation

Kinetic parameter	Limonene				*trans*-Cinnamaldehyde					
	SCL1		CL1		SCL1		SCL2		CL2	
	ND	DD	ND	DD	ND	DD	ND	DD	ND	DD
DT_50_ (days)	0.08	0.11	0.60	0.70	0.16	0.16	0.26	0.27	0.28	0.20
DT_90_ (days)	0.28	0.35	2.00	2.32	0.54	0.54	0.88	0.91	0.94	0.67
*k* (day^−1^)	8.30	6.54	1.15	0.99	4.25	4.25	2.63	2.53	2.45	3.45
*R*^2^	0.9843	0.9973	0.9968	0.9817	0.9996	0.9998	0.9979	0.9975	0.9960	0.9999

Abbreviation: *CL* clay loam soil, *DD* double dose, *DT*_*50*_ half-life time, *DT*_*90*_ 90% dissipation time, *k* rate constant, *ND* normal dose, *R*^2^ coefficients of determination, *SCL* sandy clay loam soil

**Fig. 3:**
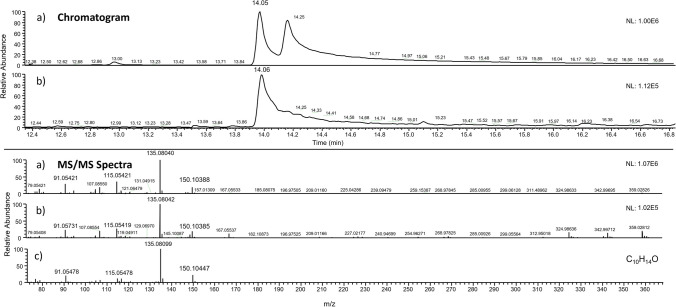
GC-Q-Orbitrap chromatogram and MS/MS spectra of **a** standard of thymol at 200 µg/L and **b** thymol (163.3 µg/kg) in sandy clay loam soil 1 at normal dose rate at day 1; **c** MS/MS spectra of thymol collected in NIST library. The theoretical molecular weight of thymol is 150.10392 *m*/*z*.

To identify potential transformation products or metabolites formed during the degradation process of limonene, an untargeted analysis (suspect and unknown modes) was performed. There are different pathways of transformation of limonene where different metabolites can be obtained such as carveol, carvone, or perillyl alcohol (van der Werf et al. 1999). For suspect analysis, these metabolites were searched using QualBrowser. For the tentative identification of them, their molecular weights and fragments collected in the literature and in the NIST library were used. However, none of the metabolites was detected using this approach. To expand the search for other metabolites, the Compound Discoverer program was used, carrying out an unknown analysis. This software allows the comparison of the molecular weights and fragments obtained in the analysis for each retention time with those collected in commercial or home-made databases. Four possible metabolites have been tentatively found: thymol, cymene, isoterpinolene, and cymenene. Thymol, as it can be seen in Fig. 3, and cymene have been confirmed with standards, obtaining a confidence level of 1 (Schymanski et al. 2014). For the quantification of isoterpinolene and cymenene, a semi-quantification was carried out, using limonene as standard. In SCL1 soil, all four metabolites were detected at both dose rates. However, isoterpinolene and cymenene were below the LOQ at normal dose rate. Metabolites were found to be present at concentrations of between 2.2 and 175.2 µg/kg at the normal dose and 16.6 to 317.3 µg/kg at the double dose (Table 3). The metabolite found at the highest concentration at the two doses was thymol (175.2 at normal dose rate and 317.3 at double dose rate). Furthermore, cymene had the lowest concentration at a double dose rate (48.7 µg/kg). In most of the detected metabolites, an initial concentration increase was observed in the first few days of the study, but it eventually decreased. For CL1 soil, only thymol was detected at both dose rates. Its concentration was lower (55.6 µg/kg) compared to that obtained in SCL1 soil (175.2 µg/kg). This could confirm that, as more microorganisms were present in the soil, more amounts and concentration of metabolites have been produced. To identify more polar metabolites, soil extracts were also analyzed by UHPLC. The data was processed with Compound Discoverer and MassChemSite programs. However, no metabolites have been detected.

**Table 3:** Concentration (µg/kg) of limonene metabolites obtained by GC-HRMS

Metabolites	Doses	0 h	4 h	1 day	1.5 day	2 days	3 days	4 days	7 days
SCL1									
Thymol	**ND**	107.5	100.2	163.3	175.2	128.5	37.5	114.5	109.3
	**DD**	317.3	127.2	163.3	179.5	120.2	187.3	68.2	146.1
Cymene	**ND**	17.1	19.7	4.8	2.2	39.3	22.5	22.9	< LOD
	**DD**	16.6	26.0	47.2	48.7	19.1	19.9	< LOD	< LOD
Isoterpinolene	**DD**	44.7	45.8	97.3	104.9	59.6	60.6	35.6	36.2
Cymenene	**DD**	58.6	59.8	98.9	100.4	61.5	61.1	50.1	51.5
CL1									
Thymol	**ND**	53.7	55.6	52.9	55.4	51.9	20.7	25.8	75.5
	**DD**	137.3	138.0	110.7	110.5	60.0	104.8	27.9	106.6

Abbreviation: *CL* clay loam soil, *DD* double dose rate, *ND* normal dose rate, *SCL* sandy clay loam soil

Toxicity Estimation Software Tool (TEST) software has been used to determine metabolite estimated and experimental toxicity (LD_50_) in rats (US EPA 2023). As can be seen in Table 4, the toxicity of the metabolites formed was very similar to limonene (4.84 g/kg), being thymol the most toxic metabolite (LD_50_ = 0.65 g/kg).

**Table 4:** LD_50_ values of limonene, *trans*-cinnamaldehyde, and their metabolites

Compound	Oral LD_50_ (g/kg)	
	Predicted	Experimental
Limonene	4.84	5.30
Thymol	0.65	0.98
Cymene	3.13	4.75
Cymenene	4.96	-
Isoterpinolene	4.41	3.65
*trans*-Cinnamaldehyde	2.36	-
Cinnamic acid	2.29	2.50
4-Hydroxycinnamc acid	2.81	-
CM1	2.87	-
CM2	1.92	-
CM3	3.87	3.60
CM4	2.53	2.00

Abbreviation: *LD*_*50*_ median lethal dose

#### *trans*-Cinnamaldehyde study

First, the degradation of *trans*-cinnamaldehyde in SCL2 and CL2 soils was studied. *trans*-Cinnamaldehyde degradation (Fig. 2) also occurred rapidly at both dose rates and soil types. In both types of soil, *trans*-cinnamaldehyde was degraded after 4 days, following a first-order kinetic. Its half-life times were 0.28 days (CL2 soil) and 0.26 days (SCL2 soil) at normal dose rate, while at double dose, they have been 0.20 days (CL2 soil) and 0.27 days (SCL2 soil). On the other hand, DT_90_ values were 0.60 days in CL1 soil and 0.08 days in SCL1 soil at the normal dose rate, and at double dose rate, they were 0.70 days in CL1 soil and 0.11 days in SCL1 soil (Table 2). As these values show, this compound degraded equally in the two soil types. In this case, the two different tested soils had a similar percent of organic matter (1.4% for CL2 soil and 1.5% for SCL2 soil). Therefore, it is understandable that it has degraded similarly in two soils. To determine whether the amount of organic matter really influences the degradation process, the same experiment was carried out using SCL1 soil (Fig. 2). This soil caused *trans*-cinnamaldehyde to degrade slightly faster and disappearing after 3 days. In this soil type, *trans*-cinnamaldehyde also followed a first-order kinetic. At the normal and double dose rate, the value of DT_50_ for *trans*-cinnamaldehyde was 0.16 days (Table 2). As expected, *trans*-cinnamaldehyde took less time to be degraded than in the other two soils containing less organic matter (SCL2 and CL2).

To perform unknown analysis, Compound Discoverer and MassChemSite software were used. When Compound Discoverer was utilized, 4-hydroxycinnamic acid and cinnamic acid have been tentatively identified. Both metabolites were found in CL2 and SCL2 soils but not in SCL1 soil. The adduct of these compounds was [M-H]^−^ with retention times of 12.15 and 12.43 min, respectively. To quantify them, a semi-quantification has been carried out using the calibration curve obtained for *trans*-cinnamaldehyde. Although both compounds appeared quickly, they also eventually degraded (Table 5). Greater amounts of 4-hydroxycinnamic acid (111.8 µg/kg) were produced than cinnamic acid (37.7 µg/kg).

**Table 5:** Concentration (µg/kg) of *trans*-cinnamaldehyde metabolites obtained by UHPLC-HRMS

Metabolites	Doses	0 h	4 h	1 day	1.5 day	2 day	3 day
SCL2							
Cinnamic acid	**ND**	37.7	32.3	30.0	25.8	25.0	< LOD
	**DD**	32.8	27.5	11.7	11.0	< LOD	< LOD
4-Hydroxycinnamic acid	**ND**	111.8	102.9	32.4	35.1	34.7	< LOD
	**DD**	127.9	140.9	12.7	12.2	< LOD	< LOD
CM1	**DD**	151.9	140.6	33.3	27.0	< LOD	< LOD
CM2	**DD**	772.7	882.3	644.0	714.7	96.5	81.4
CM3	**DD**	156.2	198.4	136.7	144.6	< LOD	< LOD
CM4	**DD**	453.6	488.9	308.9	382.4	200.1	85.8
CL2							
Cinnamic acid	**ND**	45.8	34.8	26.1	25.6	24.7	< LOD
	**DD**	42.4	36.1	12.1	11.0	< LOD	< LOD
4-Hydroxycinnamic acid	**ND**	222.1	125.0	64.5	34.5	29.8	< LOD
	**DD**	72.7	70.2	38.8	21.2	< LOD	< LOD
CM2	**DD**	725.2	666.4	716.6	710.5	20.6	11.0
CM3	**DD**	191.2	180.7	178.1	158.9	< LOD	< LOD
CM4	**DD**	404.8	402.4	436.4	402.8	79.1	54.7

Abbreviation: *CL* clay loam soil, *DD* double dose rate, *ND* normal dose rate, *SCL* sandy clay loam soil

**Fig. 4:**
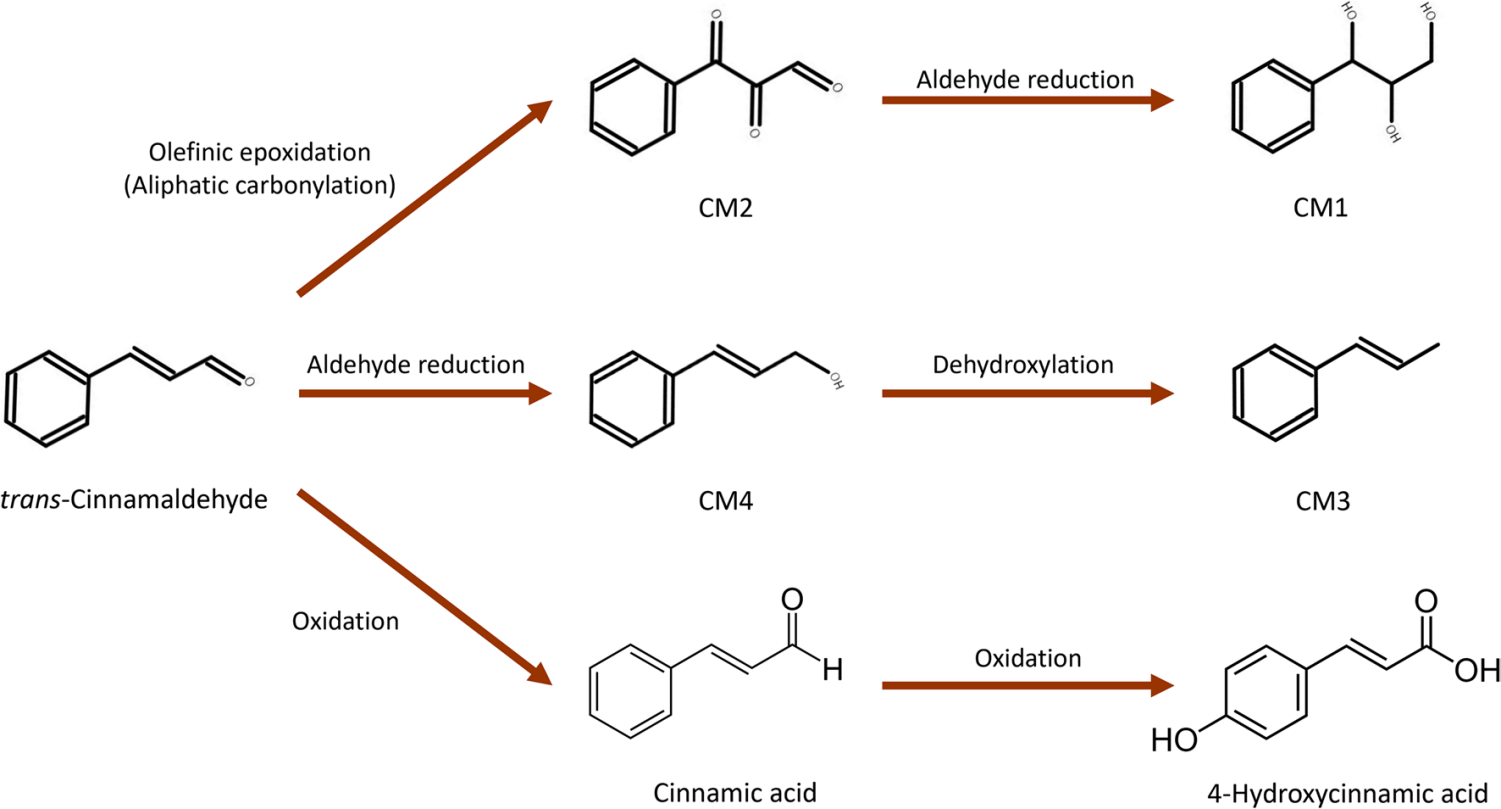
Structure of unknown metabolites of *trans*-cinnamaldehyde found with MassChemSite and Compound Discoverer.

Four other possible metabolites were tentatively found using MassChemSite program. This software allows the elucidation of possible transformation products of the precursor compound, giving data on the precursor ion of the metabolite as well as its possible structure and adduct formed. These compounds were derivatives of *trans*-cinnamaldehyde and have been named CM1, CM2, CM3, and CM4 (Fig. 4). The metabolite structures CM3 and CM4 can be related to the structure of *trans*-β-methylstyrene and cinnamyl alcohol, respectively. Some studies have evaluated the biotransformation of *trans*-cinnamaldehyde to cinnamyl alcohol and cinnamic acid by fungi such as *Mucor* (Ma et al. 2011). The degradation of *trans*-cinnamaldehyde to styrene has also been described (Balaguer et al. 2014; Becerril et al. 2019). However, CM1 and CM2 have not been described previously. Their adduct was [M+H]^+^ and their retention times were 3.12, 13.97, 14.96, and 16.05 min, respectively. The *m*/*z* and molecular formula for these compounds are shown in Table S4. These metabolites were observed only when commercial biopesticide containing cinnamon extract was applied to the double dose rate (Table 5). Furthermore, CM1 metabolite was not detected in CL2 soil. In this case, it was not possible to find these metabolites in SCL1 soil either. A semi-quantification has also been performed, using *trans*-cinnamaldehyde as standard. These four metabolites were almost completely degraded after a few days. After 2 days, CM1 and CM3 concentrations in SCL2 and CL2 soils were below the LOD. While for CM2 and CM4, there were still detected after 3 days. For SL2 soil, the concentrations (81.4–85.8 µg/kg) were higher than in the CL2 soil (11.0–54.7 µg/kg). Concentrations of these metabolites ranged between 25.0 and 882.3 µg/kg. The most highly concentrated metabolite was CM4 in both soils (SCL2 and CL2). Both CM2 and CM4 concentrations were higher than CM1 and CM3. Looking at Fig. 4**,** it can be noted that CM4 and CM2 would be intermediate steps in the formation of the other two metabolites, respectively. CM1 and CM3 may derive from these other metabolites and therefore their formation is lower.

Finally, *trans*-cinnamaldehyde (2.36 g/kg) is slightly more toxic than limonene (Table 4). Similar to limonene, the metabolites found for *trans*-cinnamaldehyde exhibited a similar level of toxicity. CM2 has a lower LD_50_ of 1.92 g/kg, and CM3 has a higher LD_50_ of 3.87 g/kg. These compounds are not highly toxic and stay in the soil for a short period of time; however, it would be necessary to monitor their presence in a real scenario to confirm their low toxicity.

## Conclusions

This study evaluated for the first time the *trans*-cinnamaldehyde degradation in different soil types. In addition, it was possible to detect several unknown metabolites produced as a result of its degradation. Limonene and *trans*-cinnamaldehyde have undergone rapid degradation in soil. Moreover, the metabolites found were also rapidly degradable compounds, resulting in no risk to the environment. These compounds and their metabolites have a high LD_50_ values; therefore, they were not highly toxic. This confirms the value of commercial biopesticides to fight against pests but not endangering the environment.

Degradation could have been mainly due to microbial action of microorganisms that are present in the soil. Using software such as Compound Discoverer or MassChemSite is a good strategy for searching for potential metabolites that are generated during this process. It would be interesting to reproduce this study in soils with different characteristics and other types of environmental and food matrices to check the matrix influence in the degradation of these products. Thus, a broader vision of these commercial biopesticides could be obtained.

### Supplementary Information

Below is the link to the electronic supplementary material.

Supplementary file1 (DOCX 25.5 KB)
