# Supplementary material for: Degradation of limonene and trans-cinnamaldehyde in soil, and detection of their metabolites by UHPLC and GC-HRMS
Source: Environ Sci Pollut Res Int. 2024 Apr 26;31(22):33058–68. doi: 10.1007/s11356-024-33334-6 (PMC11133037; doi:10.1007/s11356-024-33334-6)
Supplement: Supplementary file 1 — Supplementary file1 (DOCX 25.5 KB) [file 11356_2024_33334_MOESM1_ESM.docx]

**Degradation of limonene and *trans*-cinnamaldehyde in soil, and detection of their metabolites by UHPLC and GC-HRMS**

Alba Reyes-Ávila, Antonia Garrido Frenich, Roberto Romero-González*

Research Group “Analytical Chemistry of Contaminants”, Department of Chemistry and Physics, Research Centre for Mediterranean Intensive Agrosystems and Agrifood Biotechnology (CIAMBITAL), Agrifood Campus of International Excellence (ceiA3), University of Almeria, 04120 Almeria, Spain

*Corresponding author: [rromero@ual.es](mailto:rromero@ual.es)

**ORCID codes**

Alba Reyes Ávila: 0000-0002-0624-3754

Antonia Garrido Frenich: 0000-0002-7904-7842

Roberto Romero-González: 0000-0002-2505-2056

**Table S1.** Physicochemical characteristics of soils

| **Type of soil** | **Code** | **pH** | **Carbon Monoxide (%)** | **Organic matter (%)** | **% Grit** | **% Sand** | **% Silt** | **% Clay** |
| --- | --- | --- | --- | --- | --- | --- | --- | --- |
| Sandy clay loam | SCL1 | 8.4 | 2.4 | 4.1 | 74.2 | 51.9 | 24.3 | 23.9 |
|  | SCL2 | 8.4 | 0.9 | 1.5 | 23.0 | 58.6 | 14.2 | 27.2 |
| Clay loam | CL1 | 8.4 | 0.8 | 1.4 | 46.0 | 29.6 | 33.1 | 37.2 |
|  | CL2 | 8.6 | 0.6 | 1.4 | 12.1 | 26.1 | 44.4 | 29.5 |

**Table S2.** Characteristic chromatographic-MS parameters of limonene and *trans*-cinnamaldehyde

| **Compound** | **Molecular formula** | **Retention time (min)** | **Precursor ion** | | | **Fragment ions** | | | **Method** |
| --- | --- | --- | --- | --- | --- | --- | --- | --- | --- |
| *trans*-Cinnamaldehyde | C_9_H_8_O | 14.54 | **Adduct** | **Theorical mass (*m/z*)** | **Error mass (ppm)** | **Molecular formula** | **Theorical mass (*m/z*)** | **Error mass (ppm)** | UHPLC |
|  |  |  | [M+H]^+^ | 133.0648 | -3.393 | C_8_H_9_ | 105.0699 | 0.220 |  |
|  |  |  |  |  |  | C_7_H_7_ | 91.0542 | 2.012 |  |

| **Compound** | **Kovats retention**  **index (RI)** | **Retention time (min)** | **Characteristic ions** | | | | | | **Method** |
| --- | --- | --- | --- | --- | --- | --- | --- | --- | --- |
| Limonene | 1030 | 8.10 | **Molecular formula** | **Theorical mass (*m/z*)** | **Error mass (ppm)** | **Molecular formula** | **Theorical mass (*m/z*)** | **Error mass (ppm)** | GC |
|  |  |  | C_7_H_9_ | 93.0699 | 0.249 | C_6_H_7_ | 79.0542 | 0.293 |  |

**Table S3.** Recoveries and RSD of limonene and *trans*-cinnamaldehyde in different extraction methods^a^

| **Method extraction** | **Compound** | **Recovery (%)** | **RSD (%)** |
| --- | --- | --- | --- |
| Rotatory extraction  30 min | Limonene | 56.7 | 5.6 |
|  | *trans*-Cinnamaldehyde | 50.9 | 2.2 |
| Rotatory extraction  60 min | Limonene | 98.5 | 4.7 |
|  | *trans*-Cinnamaldehyde | 101.4 | 1.0 |
| UAE  20 min | Limonene | 111.6 | 4.6 |
|  | *trans*-Cinnamaldehyde | 111.4 | 2.7 |

^a^Abbreviation: RSD: relative standard deviation; UAE: ultrasound-assisted extraction

**Table S4.** UHPLC-Q-Orbitrap parameters of *trans*-cinnamaldehyde metabolites found with MassChemSite

| **Code** | **Retention time (min)** | **Molecular formula** | **Adduct** | ***m/z*** |
| --- | --- | --- | --- | --- |
| CM1 | 3.12 | C_9_H_12_O_3_ | [M+H]^+^ | 169.0859 |
| CM2 | 13.97 | C_9_H_6_O_3_ | [M+H]^+^ | 163.0390 |
| CM3 | 14.96 | C_9_H_10_ | [M+H]^+^ | 119.0855 |
| CM4 | 16.05 | C_9_H_10_O | [M+H]^+^ | 135.0804 |
